# Abdominal Epilepsy: A Rare Cause of Unexplained Abdominal Pain

**DOI:** 10.7759/cureus.10120

**Published:** 2020-08-29

**Authors:** Anvesh Balabhadra, Apoorva Malipeddi, Niloufer Ali, Raju Balabhadra

**Affiliations:** 1 Department of Neurology, Gandhi Medical College and Hospital, Hyderabad, IND; 2 Department of Internal Medicine, Gandhi Medical College and Hospital, Hyderabad, IND; 3 Department of Neurology, Aster Prime Hospital, Hyderabad, IND; 4 Department of Neurological Surgery, Aster Prime Hospital, Hyderabad, IND

**Keywords:** abdominal epilepsy, temporal lobe epilepsy, unexplained abdominal pain, eeg

## Abstract

Abdominal epilepsy (AE) is a very rare diagnosis; it is considered to be a category of temporal lobe epilepsies and is more commonly a diagnosis of exclusion. Demographic presentation of AE is usually in the pediatric age group. However, there is recorded documentation of its occurrence even in adults. AE can present with unexplained, relentless, and recurrent gastrointestinal symptoms such as paroxysmal pain, nausea, bloating, and diarrhoea that improve with antiepileptic therapy. It is commonly linked with electroencephalography (EEG) changes in the temporal lobes along with symptoms that reflect the involvement of the central nervous system (CNS) such as altered consciousness, confusion, and lethargy. Due to the vague nature of these symptoms, there is a high chance of misdiagnosing a patient. We present the case of a 20-year-old man with AE who was misdiagnosed with psychogenic abdominal pain after undergoing multiple investigations with various hospital departments.

## Introduction

Abdominal epilepsy (AE) is a rare syndrome, even rarer when seen in adults and presents with paroxysmal symptoms favouring an abdominal pathology that result from seizure activity [1]. The spectrum of AE is characterized by (a) otherwise unexplained, paroxysmal gastrointestinal complaints; (b) symptoms suggesting central nervous system (CNS) disturbance; (c) abnormal electroencephalography (EEG) with findings specific for a seizure disorder; and (d) improvement of symptoms with anticonvulsant therapy [2]. A review of the history of this syndrome has yielded 36 cases reported in the English literature in the previous 34 years [3]. Due to the paucity of reported cases, there is a pressing need for accurate diagnosis by increasing awareness among physicians in order to avoid misinterpretation of these symptoms as "functional" or "psychogenic" [4]. This case report was previously presented as an abstract at the 144th Annual Meeting American Neurological Association [5].

## Case presentation

The patient is a 20-year-old right-handed male. He presented to the General Surgery Clinic with recurrent episodes of severe "piercing" epigastric abdominal pain for six months. These episodes were associated with non-projectile vomiting followed by lethargy or loss of consciousness which lasted for a "few minutes" after each episode. They were neither associated with food intake nor had any aggravating or relieving factors. Each episode of abdominal pain lasted for 8-12 minutes and occurred three to five times per day. They were sudden in onset and resolved spontaneously. His birth, developmental, and past medical histories were unremarkable.

General and local examinations of the abdomen and nervous system did not reveal any significant findings. He was prescribed analgesics and proton pump inhibitors and he was reviewed after one week. He did not report any significant improvement in his symptoms. His complete blood picture, stool for ova and parasites, 24 hr Holter ECG, ultrasound abdomen and CECT abdomen were unremarkable.

Following this visit, he was put in for a gastroenterology referral; upper gastrointestinal (GI) endoscopy and barium swallow studies were found to be normal after which he was referred to the psychiatrist in view of psychogenic abdominal pain. There was no psychiatric intervention and he was referred to the neurology department for a second opinion.

**Figure 1:**
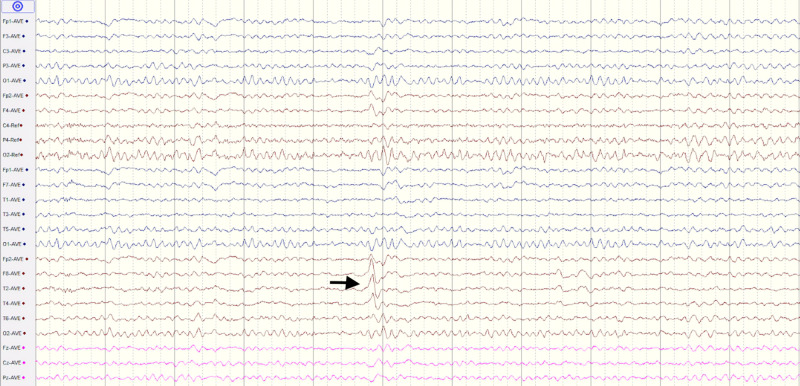
EEG findings of a 20-year-old male patient with abdominal epilepsy 30-Minute awake EEG showing sharp wave discharges in the right temporal leads. EEG = electroencephalography.

MRI brain was found to be normal. At this point, an epileptic aetiology was suspected. As in many Indian settings, the facilities for an ambulatory-EEG or video-EEG are either unavailable or beyond the affordability of most patients, a 30-minute awake EEG was ordered which revealed an abnormality in the form of repetitive sharp waves in the right temporal leads which were highly suggestive of an epileptogenic focus arising from the right temporal lobe as seen in Figure 1.

 

Then, the conclusive diagnosis of AE was made and he was started on oxcarbazepine 450mg BID. He was scheduled with regular monthly follow-up visits and was compliant with his medication. A follow-up over a 12-month period showed a progressive decrease in seizure frequency and has been asymptomatic for the past four months. 

## Discussion

AE is a rare disorder and considering the vague nature of its symptoms, it is usually unconsidered and often misdiagnosed or missed from being diagnosed. It commonly occurs in the pediatric age group, but there is also documentation of its occurrence in adults [6]. It is characterized by otherwise unexplained, paroxysmal GI complaints, symptoms of a CNS disturbance, an abnormal EEG report with findings specific for a seizure disorder and improvement with anticonvulsant medication [3].

AE has a variety of presenting symptoms. GI symptoms include paroxysmal pain, nausea, bloating, and diarrhoea, whereas the CNS symptoms can comprise of dizziness, lethargy, headache, confusion, syncope, and transient blindness [3,7]. As AE is also a type of autonomic epilepsy, it can also be associated with some autonomic phenomena co-relating with the episodes such as pallor or cold sweating [8]. Abdominal auras can also be seen with manual and oral automatisms, that is, an auto motor seizure [9]. 

Although many mechanisms have been outlined, the cause of AE still remains unclear. We hypothesise that the Sylvian fissure and insular cortex lying right beneath it could be the origin of the seizure as they coincide with the locations of the abdomen on the Sensory homunculus. Additionally, the M2 portion of the middle cerebral artery courses through the Sylvian fissure [10]. Any pathology of the vessel, particularly at this segment could be assumed to play a role in epilepsies of the temporoparietal lobes. Phan et al. reported a case of ictal abdominal pain associated with parietal lobe haemorrhage and proposed the role of the somatosensory area I in pain perception [11]. There have been previously reported cases of ictal abdominal pain that have been associated with right parietooccipital encephalomalacia, biparietal atrophy and bilateral perisylvian polymicrogyria [12,13].

Recurrent abdominal pain may also be seen in visceral hyperalgesia, peptic ulcer disease and abdominal migraine [14,15]. The most common differential diagnosis for AE is abdominal migraine as they have many overlapping features. Duration of the symptoms may be used to differentiate the two entities; the duration is longer in abdominal migraine than in AE. The EEG is usually abnormal in AE and may be used to confirm the diagnosis of AE [6].

Patients with AE may have the following possible variations in the EEGs:

(1) Patients may have normal EEG patters in the inter-ictal periods and diagnosis must not be purely based on an EEG [15].

(2) Extra-temporal origins of epileptic foci; secondary generalisation.

(3) There are strong suggestions that EEG conducted after the first 24 hours after the epileptic episode can detect abnormalities to a greater extent [16].

## Conclusions

This case clearly exhibits how coming to a conclusive diagnosis of AE can be challenging and time-consuming as the symptomatology is indistinct and as the suspicion is low especially in the case of adults. As AE might often be overlooked or misdiagnosed, it should be taken into account in patients presenting with episodic, recurrent and paroxysmal GI complaints along with symptoms suggestive of CNS disturbance which do not improve with standard treatment modalities and after thoroughly excluding the more common causes in order to provide and an accurate and timely diagnosis. We must also be aware of the stigma that revolves around the diagnosis of a somatoform disorder in these scenarios and quickly consider getting an EEG, preferably a video-EEG if the facilities are available for such patients so that their symptoms are not falsely labelled as "psychogenic" or "functional". If the EEG findings are abnormal, the treatment involves the initiation of antiepileptic drugs with regular follow-up. 450mg oxcarbazepine BID was very effective in the management of AE for our patient.
